# Comparison of Rehabilitative Interventions That Ameliorate Post-stroke Working Memory Deficit: A Systematic Review

**DOI:** 10.7759/cureus.30014

**Published:** 2022-10-06

**Authors:** Lakshmi Sai Deepak Reddy Velugoti, Godfrey Tabowei, Greeshma N Gaddipati, Maria Mukhtar, Mohammed J Alzubaidee, Raga Sruthi Dwarampudi, Sheena Mathew, Sumahitha Bichenapally, Vahe Khachatryan, Asmaa Muazzam, Chandani Hamal, Lubna Mohammed

**Affiliations:** 1 Department of Neurology, California Institute of Behavioral Neurosciences & Psychology, Fairfield, USA; 2 Department of Internal Medicine, California Institute of Behavioral Neurosciences & Psychology, Fairfield, USA; 3 Department of Research, California Institute of Behavioral Neurosciences & Psychology, Fairfield, USA; 4 Department of Pathology Research, California Institute of Behavioral Neurosciences & Psychology, Fairfield, USA; 5 Department of Internal Medicine/Family Medicine, California Institute of Behavioral Neurosciences & Psychology, Fairfield, USA

**Keywords:** post stroke, comparision, multimodal rehabilitation, goal setting, physical activity, computer assisted cognitive rehabilitation, transcranial direct-current stimulation, stroke, working memory deficits

## Abstract

Stroke is one of the most common causes of disability in the world. It has sensory, motor, and cognitive symptoms. Many cognitive domains might get involved in a stroke. This systematic review focuses on working memory domain deficits after stroke and their various rehabilitation methods. This review is based on Preferred Reporting Items for Systematic Reviews and Meta-Analyses(PRISMA) guidelines. For this review, we have searched PubMed, Google Scholar, and Science Direct databases and screened thoroughly with the inclusion criteria of free full-text English papers in the last 10 years that have exclusively studied humans. The articles included in the search are randomized control trials (RCTs), observational studies, meta-analysis studies, systematic reviews, and traditional reviews. Consequent quality assessment was done using the most commonly used tools for each type of study and eight papers were selected. From these papers, full-text articles were studied, analyzed, and tabulated. We found five different rehabilitation methods: transcranial direct-current stimulation, computer-assisted cognitive rehabilitation, physical activity, goal setting, and multimodal rehabilitation. We found that goal setting, computer-assisted cognitive rehabilitation, and multimodal rehabilitation can improve working memory deficits. While transcranial direct current stimulation and physical activity were inconsistent, further studies are needed. The small sample size, no follow-up, the inclusion of only a few studies, the size of the stroke, and comorbid conditions like mild cognitive impairment, dementia, and depression were the main limitations of this study. Future reviews must include a larger number of studies with large sample sizes, including follow-up as an inclusion criterion. We need more clinical trials on these methods for better knowledge.

## Introduction and background

Stroke is the second most common cause of dementia and the third most common cause of morbidity [1]. It can occur due to ischemic (most common) or hemorrhagic causes and can decrease the blood supply of the brain. The most common involved symptoms besides physical deficits (such as complete or partial paralysis, sensory loss, and altered sensations) are dysfunction in learning, memory, and executive functions. This affects nearly 83% of post-stroke patients with cognitive dysfunction, and more than 60% of stroke survivors report cognitive dysfunction, even for up to 10 years [2]. While motor and sensory deficits can affect patients’ quality of life and economic status, memory deficit will affect patients’ daily activities, their profession, and their families. Although the motor and cognitive functions are fundamentally treated as well-defined and separate entities while treating and diagnosing, both entities play an essential role in the post-stroke assessment of behavior and disability [3]. When it comes to cognitive function, working memory has a fundamental role in performing complex behavior [4]. Damage to working memory function causes a drop in the complex cognitive function of the brain to perform everyday activities such as memorization, communication, planning, reading, and writing [5].

What is working memory? It is defined as a multi-component system involved in goal-directed behavior that involves retaining and manipulating information [6]. In simple terms, it is explained as a “sketchpad of conscious thought” [7]. for example, problem-solving in our mind and navigating to our home within our mind. Baddeley and Hitch described it as an essential model with four sub-components arranged hierarchically [6]. They are the phonological loop, visuospatial sketchpad, central executive, and episodic buffer [6]. The central executive supervises the other three subcomponents to store visual, spatial, and phonological information [8]. Working memory reaches its optimal capacity by 20 to 25 years of age. Then each subcomponent declines slowly with further aging at a different rate, which is explained by visual memory declining faster than phonological memory [9]. Many previous imaging studies found that working memory is a part of the lateral prefrontal and parietal cortex of the brain [10]. These areas are supplied by the middle and anterior cerebral artery branches and later drained into cerebral venous sinuses. The stroke affecting these regions will impair the functions. 

Though post-stroke dementia, episodic memory, and long-term memory deficits after stroke have been studied abundantly, working memory deficits after stroke have less information. There is ongoing research on various methods to improve working memory skills and reduce the patient’s morbidity on the efficacy of each rehabilitation. To our knowledge, there is a lack of information on the comparison and efficacies of each intervention; such information is vital for selecting an effective intervention. This review article compares rehabilitative interventions to reduce the working memory deficit after stroke.

## Review

Methods

This systematic review was conducted based on the Preferred Reporting Items for Systematic Reviews and Meta-Analyses (PRISMA) 2020 guidelines [11].

Search Strategies

 We performed this systematic review using PubMed, Google Scholar, and Science Direct databases with keywords “stroke” and “working memory deficits” in all three databases, including Medical Subject Headings (MeSH) terms and keywords in PubMed search, and included multiple filters for each journal. These details are illustrated in Table 1.

**Table 1:** Databases used, keywords, search strategy, and filters applied

Databases	Keywords	Search strategy	Filters applied	Search results
PubMed	Stroke, Working memory deficit	Stroke OR "Stroke/psychology"[Majr]) AND Working memory deficit OR Short term memory loss OR ("Memory, Short-Term/drug effects"[Majr])	1. Free Full text, 2. Last ten years, 3. Humans, 4. English, 5. Article type, a) Randomized control trials (RCT,) b) Observational studies, c) Meta-analysis, d) Systematic review, e) Traditional Review	106 (Last searched on May 22, 2022)
Google Scholar	Stroke, Working memory deficit	“Stroke” AND “Working memory deficit intervention”	Publications from 2012 to 2022; Screened first 300 articles	17,800 (last searched on May 22, 2022)
Science Direct	Stroke, Working memory deficit	“Stroke” AND “Working memory”	Publications from 2012 to 2022	2,382 (last searched on May 22, 2022)

Inclusion and Exclusion Criteria

We have included free, full-text English papers that have exclusively studied humans in the last 10 years. The articles included are randomized control trials (RCTs), observational studies, meta-analysis studies, systematic reviews, and reviews.

Selection Strategy

Two reviewers selected the articles independently using the same search strategy in all three databases. At first, articles were screened from the title of articles and abstracts and then later by reading full-text articles. If contradicting results regarding the article’s eligibility occurred, reviewers assessed the full-text article until the group reached a consensus.

Data Collection, Items, Analyses, and Outcome Assessment

Two reviewers collected data independently, and if reviewers found contradicting results, a full-text article was scrutinized and discussed until all reviewers reached a consensus. Collected data were analyzed and tabulated under various headings such as (i) first author and year, (ii) population along with dropout patients (for RCTs)/number of studies included (for systematic review, traditional review, and meta-analysis, (iii) intervention, (iv) duration of intervention, (v) outcome measures and assessment, (vi) follow-up assessment, and (vii) funding sources. Due to heterogeneity in the population, different interventions, and unique outcomes of each study when compared with other studies, it was not possible to perform the meta-analysis.

Risk of Bias Assessment

Each selected study was assessed for risk of bias by two reviewers independently using commonly used tools for each type of study, and only studies that scored more significant than 70% were included in this review. Table 2 shows the quality assessment tools used for each type of study and accepted studies.

**Table 2:** Risk of bias assessment

Quality assessment tool	Type of study	Total score	Accepted score (>70%)	Accepted studies
Cochrane collaboration risk-of-bias tool (CCRBT)	Randomized Control Trial	7	5	Fishman et al. [12]. Studer et al. [13]. Park et al. [14]. Liu-Ambrose et al [15]. Bunketorp-Käll et al. [16]
Preferred reporting items for systematic reviews and meta-analyses (PRISMA) 2020	Systematic Review	44	31	Van de Ven et al. [17]
	Meta-analysis			Oberlin et al. [2]
Scale for the assessment of narrative review articles (SANRA)	Traditional Review	12	9	Madhavan et al. [18]

Results

**Figure 1:**
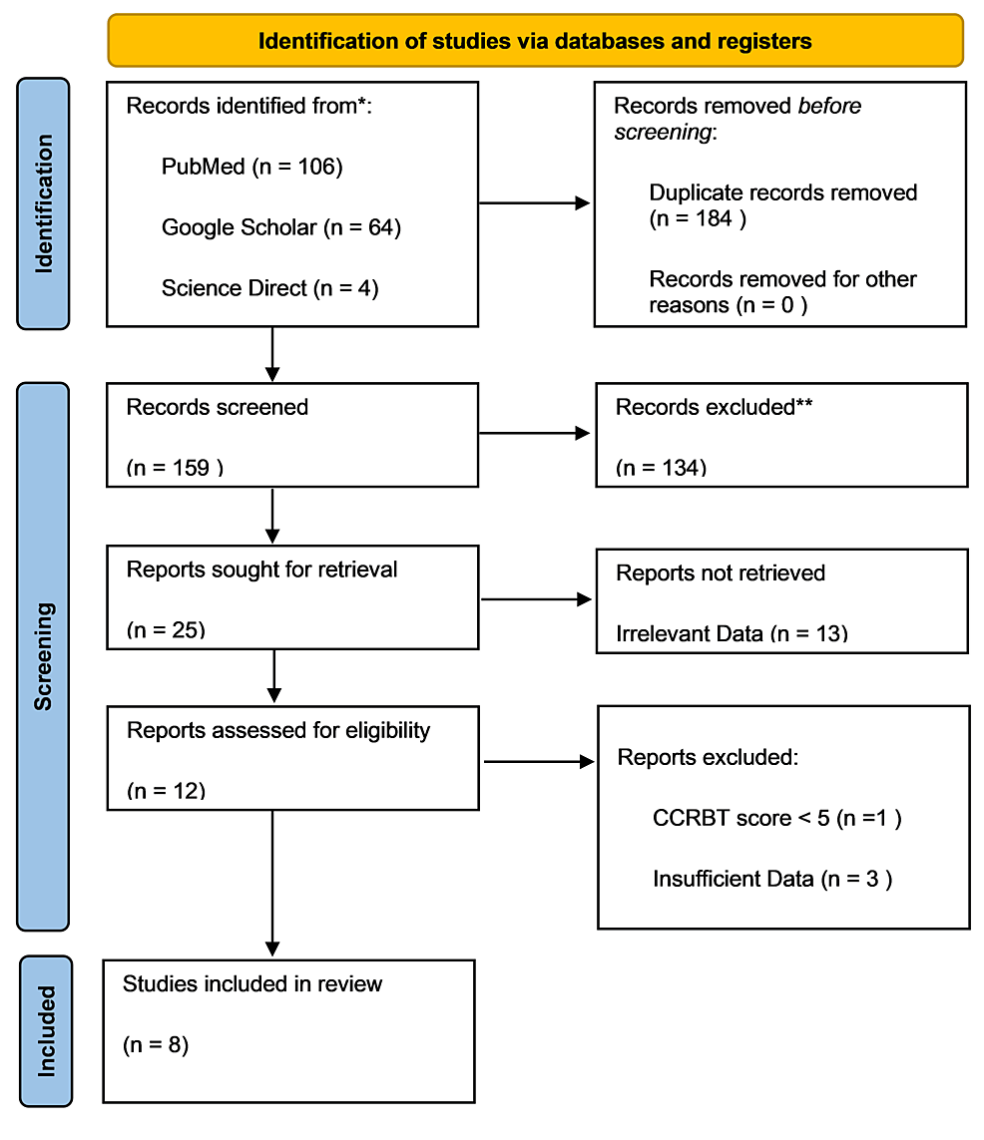
Flowchart of the study selection process CCRBT: Cochrane collaboration risk-of-bias tool

There were 174 potentially related titles found in the database search. One hundred and fifty-nine records were kept after duplicates were removed. When the titles and abstracts of these records were evaluated based on the qualifying criteria for this review, 25 articles remained. From these, 13 reports were discarded because of irrelevant data. Eight papers with a score of more than 70% were allowed in the review after each publication underwent a quality assessment. There were four RCTs, one traditional review, one meta-analysis, one systematic review, and one pilot research. A flow diagram of the study selection and screening process is shown in Figure 1.

Study Characteristics

The main characteristics of the clinical trials, pilot study, meta-analysis, traditional review, and systematic review are shown in Tables 3, 4 respectively. Of these eight studies, one pilot study and one traditional review used transcranial direct current stimulation intervention, one systematic review and the same pilot study used computer-assisted cognitive rehabilitation (CACR) intervention, one RCT and one meta-analysis focussed on physical activity as intervention, one RCT applied goal setting as intervention, and one RCT adopted multimodal rehabilitation with follow-up. All these articles assessed the working memory domain using different outcome measures.

**Table 3:** Study characteristics of the pilot study and clinical trials tDCS: transcranial direct current stimulation; CPT: continuous performance test; RCT: randomized control trial; WSST: Wechsler Spatial Span Test; R-MT: rhythm and music therapy; H-RT: horse riding therapy

Study	Type of study	Population	The number of studies included	Intervention	Duration of training	Results	Follow-up	Funding sources
Park et al. 2013 [14]	Pilot study	Newly diagnosed with stroke	11 (0)	Simultaneous use of tDCS with the computer-assisted cognitive rehabilitation	Using tDCS stimulator with two mA intensity for 30 minutes on the bilateral prefrontal cortex (anode) and non-dominant arm (cathode) and Korean computer-assisted cognitive rehabilitation program for 30 minutes a day, five times a week for a mean period of 18.5 days.	There is no significant difference between the intervention and control group except in auditory CPT and Visual CPT in the pre/post ratio between the intervention and control group.	No follow-up	Supported by a grant from the Biomedical Research Institute of Chonbuk National University Hospital
Studer et al. 2021 [13]	RCT	Adult stroke patients with visuospatial memory impairments	95(12)	Wizard memory game, a tablet-based training for visuospatial working memory	Both intervention (patients with precommitment) and the control group were asked for self-directed training for 30 minutes a day for two weeks in addition to standard therapy	Primary analysis shows no statistically significant difference between the precommitment group and the control group in WSST backward. Secondary analysis dividing patients trained with wizard game had shown improvement than patients who were untrained with wizard game in WSST backward.	No follow-up	Funded by Mauritius Hospital Meerbusch
Liu-Ambrose and Eng 2015 [15]	RCT	Chronic single stroke patients (≥12 months after stroke)	28(4)	Exercise training and recreational sessions	Six months of community programs that include two exercise training sessions, one recreational session, and one leisure activity session per week for the intervention group	A 43% improvement is seen in the intervention group compared with the delayed intervention group a six-month intervention period using the difference between verbal digit forward test and the verbal digit backward test.	No follow-up	Funded by Canadian Stroke Network
Fishman, et al. 2021 [12]	RCT	Chronic stroke patients (≥3 months after stroke)	72 (0)	Goal setting	The intervention group was instructed to set a goal to improve their performance, and the control group was given standard therapy.	Improvement in digit span sequencing and digit span total was seen with goal setting.	No follow-up	Supported by Natural Sciences and Engineering Research Council Postgraduate Doctoral Scholarship and Canadian Psychosocial Association.
Bunketorp-Käll et al. 2017 [16]	RCT	Patients who had stroke ≥10 months back and ≤5 years before enrollment in this study	123 (8)	Multi-modal	Two intervention groups (one is allocated to R-MT, and the other is H-RT) and a control group allocated to rhythm and music therapy one year after the intervention	No improvement is found immediately at the post-intervention letter number sequencing test.	R-MT group has improved in letter-number sequencing of working memory while H-RT group has no statistical significance at six months follow-up	It was funded by Sten A Olsson Foundation for Research and Culture, Swedish Brain Foundation, the Swedish Stroke Association, and others.

**Table 4:** Study characteristics of the traditional review, systematic review, and meta-analysis tDCS: transcranial direct current stimulation; NIH: National Institutes of Health; WM: working memory

Study	Type of study	Population	The number of studies included	Intervention	Duration of training	Results	Follow-up	Funding sources
Madhavan and Shah 2012 [18]	Narrative Review	Stroke affected	12 studies	tDCS	Typically, a current intensity of 0.5-2 mA for a 5-20 minute period, providing a current density of 0.02-1 mA/cm^2^ and a total charge of 15-100 micro coulombs/cm^2^. Different studies used different values within this range.	Improvement in the two back letter memory task is found.	No follow-up	Not reported
van de Ven et al. 2016 [17]	Systematic Review	Adults who have suffered from stroke or acquired traumatic brain injury	20 studies	Computer-assisted cognitive rehabilitation	Cogmed training for 30-40 minutes a day five times a week for five weeks for exclusive working memory training studies Rehacom training for both attention and working memory training for variable time in other studies	WM training studies have improved subjective and objective cognitive functions. This training enhanced only verbal WM but not visual WM. In both attention and WM combined training studies, no consistent improvements were found. Only immediate short-term improvements were seen.	No follow-up	Funded by Netherlands Initiative Brain and Cognition, a part of the Organization for Scientific Research
Oberlin et al. 2017 [2]	Meta-analysis	Stroke patients ≥ 18 years old	14 studies	Physical activity (aerobic exercise, resistance therapy, or physiotherapy)	More than four weeks of physical activity	WM domain has no statistical significance	No follow-up	Funded by the NIH Training Grant

Outcome Measures

Working memory is usually measured using the digit span, letter-number sequencing, Wechsler spatial span, and visual cognitive performance tests. Each article used one or more of these tests. Though these tests help assess improvement in working memory, much more ecologically valid measures are required to evaluate real-life situations.

Discussion

In this systematic review, we aimed to compare the efficacies of rehabilitation that reduce working memory deficits after stroke. We selected five randomized control trials, two systematic reviews, and one traditional review and collectively showed five different rehabilitation methods to improve working memory. For better understanding, all included papers were divided into rehabilitation methods and discussed in detail.

Transcranial Direct Current Stimulation (tDCS)

In our review, two papers were included regarding transcranial direct stimulation of the brain. Although tDCS has the advantage of being a non-invasive procedure that delivers a low-intensity direct current through the scalp, it still has areas to hone itself because of the presence of variables from person to person, such as conductivity, the resistance of the scalp, and the dosage of current. Though there are many types of tDCS, Anodal tDCS is the most commonly used and it has significant evidence from many publications that enhance working memory when stimulated by the dorsolateral prefrontal cortex (DLPFC) [18].

In healthy individuals, anodal tDCS has increased correct responses and fewer errors were noted when compared to other types of tDCS such as cathodal or sham tDCS [18]. Some studies described that anodal tDCS promotes neural plasticity in chronic stroke survivors, which were shown as alpha waves in the electroencephalography (EEG) [19]. These alpha waves indicate functional activity of working memory along with attention, motor learning, and performance and are considered a biomarker of functional activity of the brain [19,20]. Anodal tDCS application is based on the inter-hemispheric imbalance of excitability, which is explained by hyperexcited non-lesioned DLPFC on one hemisphere of the brain because of the loss of transcallosal inhibition from lesioned DLPFC on another hemisphere of the brain [18,20]. This imbalance in excitability is a poor functional recovery marker and is corrected by applying tDCS either with anodal stimulation, which causes an excitatory stimulation on the affected hemisphere, or cathodal stimulation, which causes an inhibitory stimulation on the non-affected hemisphere and brings back the balance in both hemispheres, which enhances recovery [18]. One pilot study describes that there is a synergistic effect when tDCS combined with CACR has shown short-term improvement in the verbal two-back working memory test [14]. But, due to the small number of patients (n=11) within the trial, this study cannot be generalized to the population, and there is no evidence of any improvements in tDCS in long-term rehabilitation.

Computer-Assisted Rehabilitation

In our review, a systematic review of computer-based cognitive training included four studies only on working memory improvement after computer-based rehabilitation training using Cogmed training (Cogmed, Stockholm, Sweden) either at a rehabilitation center (three studies) or at home (one study) [17]. It is a computer program that includes audio and visuospatial tasks and always requires a motor response. Patients were trained for 30 to 40 min for five days a week for a five-week duration [17]. This training improved working memory even after three months of rehabilitation [17]. Along with working memory, objective improvements such as attention, and subjective improvements such as mood were seen to improve [17]. This systematic review also included seven studies on combined training of working memory and attention, which showed improvement in all seven studies. Still, these results are based on one or two tasks. In contrast to only working memory studies, this combined training showed only immediate recall, and only one out of seven studies showed improvement in mood. Though this is an extensively reviewed article, the lack of a proper control group and the lack of long-term evaluation of this training are the main limitations that should be addressed in future studies [17].

An RCT of 83 patients with the help of a computer game called Wizard memory game (Peak, Manchester, United Kingdom) showed a significant improvement in working memory [13]. It is a game with working memory tasks, and patients were divided into a pre-commitment group and a control group. The precommitment group was given a choice restricting option of visitor ban or continuous physician surveillance to ensure voluntary modification of one's choice to enhance the likelihood of reaching the target (decisional neuroscience) [13]. Decisional neuroscience encourages the patients to reset their behavior and promotes motivation to change behavior [13]. Precommitment and control groups are given a different frequency of training every second and fifth day of self-directed training for two weeks [13]. When comparing pre and post-changes in both verbal and visuospatial working memory, the statistical analyses have a positive correlation with the Wizard memory game. More improvements in working memory were found when this rehabilitation was used as an add-on to standard post-stroke neurorehabilitation. Thus computer-assisted decisional neuroscientific intervention promotes working memory improvement in patients when given as an add-on to standard therapy. Though this study has a proper sample size, adequate power, and randomization, follow-up studies are necessary for assessing working memory in everyday life and the sustainability of training.

Physical Activity

A six-month randomized control trial of 28 stroke survivors who had an episode ≥ one-year onset and had completed their rehabilitation was divided into an intervention group and a control group. While the intervention group received two sessions of exercise training and one session of recreation and leisure activity, the control group received usual care. The intervention group was found to have a 43% improvement in working memory [15]. The effect is dependent on the duration and type of training (aerobic or multimodal exercise training) involved [15,21]. Although very few studies had shown improvement in working memory [15], many studies had contradicting results to support this statement [1,2]. Some studies have shown improvement when combined cognitive and exercise interventions are included [22]. Because of the small sample size and other contradicting studies, further extensive sample studies are needed.

Unlike the single RCT, we also included a meta-analysis of randomized control trials for the effect of physical activity on post-stroke working memory deficits [2]. This meta-analysis included around 14 studies, but only five out of 14 studies had working memory as an outcome [2]. This study did not reach a statistical significance on working memory to support physical activity as an intervention [2].

Both studies included in this review have a small sample, not distributed evenly, worked only in the chronic phase after stroke, and one study has not shown statistical significance for the working memory domain [15,2]. Though this might not help us decide with the present studies, other cognitive domains were included along with working memory to study in these studies. Along with physical activity, domains such as selective attention, set-shifting, processing speed measures, conflict resolution, and mood are included in their outcome. Some of these outcomes have positive effects with statistical significance. Physical activities such as aerobic and recreational activities play a significant role in the development of these cognitive domains.

Goal Setting

National Clinical Guidelines promote goal setting as an essential aspect of stroke rehabilitation. Many studies have shown improvement in patients' confidence and motivation to work on rehabilitation with prior goal setting to improve motor and cognitive skills [23,24]. There are some different goal perspectives between patients and hospital staff that impede the development of the patient. The patient goal is usually long-term oriented, returning to normal and vaguely described. In contrast, the hospital staff goal is short-term, specifically focused on impairment, and motivated by financial and organizational pressures [23]. Terminating these barriers with open discussion of the goal with the patient and their families and positively encouraging and promoting the environment created by staff enabled many patients to participate in the rehabilitation [23] actively. There are tools used for goal setting, such as Return to Work (RTW) goal working sheet by American Stroke Association and goal management training [12], and can also be created and tailored per patients' requirements. Integrating well-defined, achievable goals into the goal and working on them daily will motivate the patient to work on them. We included a RCT of goal setting and its effect on stroke survivors with accurate power and a good sample size. This trial showed improvement in the intervention group [12]. It is not attributable to age, education, time since stroke, sleep, depression, or vascular risk factors after multiple corrections using SPSS statistical software (IBM Corp., Armonk, New York, United States). This signifies the importance of ‘goal setting’ as a tool that covers a broad spectrum of various stroke survivors. While the trial has given promising results in rehabilitation, it did not have a follow-up and did not include aphasia and dementia patients. Further studies could provide a better opinion when including a follow-up and inclusion of aphasia and dementia patients.

Multimodal Rehabilitation

Multimodal rehabilitations promote patients to have a wide range of functions to help with motor, sensory and cognitive functions. Because of multiple different approaches simultaneously, these can have an additive or synergistic effect. Also, some studies show an enriched environment promotes recovery with better results than being alone and socially inactive. A RCT was included in our studies, which had one control group and two intervention groups in which one had rhythm and music therapy (R-MT) and the other had dance and horse riding therapy (H-RT) for 12 weeks with two sessions weekly [16]. Many outcomes, including working memory, were statistically analyzed. It was found that the R-MT group improved working memory, even after six months of rehabilitation. Other groups did not have statistical significance to provide a proper result. Unlike all the prior rehabilitation methods, this had a good follow-up and promising results that show the retention of improvements even after rehabilitation. This study had a small strength of participants from a single region. So, a broader study region with a better number of participants can help us better understand how to incorporate a multimodal approach into clinical settings.

Limitations

This study has several limitations such as the size of the stroke and comorbid conditions like mild cognitive impairment, dementia, and depression. Along with these factors, other major limitations are most of the studies included has a small sample size, no follow-up, and only mentioned their use in the chronic phase of stroke, so they cannot be generalized to the overall population. Some of these methods have not yet been thoroughly studied. Hence future studies on these methods with RCTs would benefit us to know the benefits and side effects of these methods.

## Conclusions

There are several individual studies on each cognitive rehabilitation method, but to our knowledge, we have not found any study on the comparison of one with another rehabilitation method. We found five different rehabilitation methods in these studies, in which goal setting, CACR, and multimodal rehabilitation can improve working memory deficits. tDCS and physical activity were inconsistent and further studies are needed. From this review, the multifaceted approach with synergistic effects is seen to be the best approach because one method's disadvantage can be counterbalanced with another rehabilitation method. This systematic review has a few limitationsa, such as small number of studies included, small sample size in a few papers, and availability of follow-up only in two studies. Future studies must include large sample sizes and follow-ups as inclusion criteria. More RCTs are needed on these methods for better knowledge.

## References

[1] Unibaso-Markaida I, Iraurgi I, Ortiz-Marqués N, Amayra I, Martínez-Rodríguez S (2019). Effect of the Wii Sports Resort on the improvement in attention, processing speed and working memory in moderate stroke. J Neuroeng Rehabil.

[2] Oberlin LE, Waiwood AM, Cumming TB, Marsland AL, Bernhardt J, Erickson KI (2017). Effects of physical activity on poststroke cognitive function: a meta-analysis of randomized controlled trials. Stroke.

[3] Einstad MS, Saltvedt I, Lydersen S (2021). Associations between post-stroke motor and cognitive function: a cross-sectional study. BMC Geriatr.

[4] Lindeløv JK, Overgaard R, Overgaard M (2017). Improving working memory performance in brain-injured patients using hypnotic suggestion. Brain.

[5] Xu JJ, Ren M, Zhao JJ (2020). Effectiveness of theta and gamma electroacupuncture for post-stroke patients on working memory and electrophysiology: study protocol for a double-center, randomized, patient- and assessor-blinded, sham-controlled, parallel, clinical trial. Trials.

[6] Lugtmeijer S, Lammers NA, de Haan EH, de Leeuw FE, Kessels RP (2021). Post-stroke working memory dysfunction: a meta-analysis and systematic review. Neuropsychol Rev.

[7] Miller EK, Lundqvist M, Bastos AM (2018). Working memory 2.0. Neuron.

[8] Baddeley A (2012). Working memory: theories, models, and controversies. Annu Rev Psychol.

[9] Froudist-Walsh S, López-Barroso D, José Torres-Prioris M, Croxson PL, Berthier ML (2018). Plasticity in the working memory system: life span changes and response to injury. Neuroscientist.

[10] Nord CL, Popa T, Smith E (2019). The effect of frontoparietal paired associative stimulation on decision-making and working memory. Cortex.

[11] Page MJ, Moher D, Bossuyt PM (2021). PRISMA 2020 explanation and elaboration: updated guidance and exemplars for reporting systematic reviews. BMJ.

[12] Fishman KN, Ashbaugh AR, Swartz RH (2021). Goal setting improves cognitive performance in a randomized trial of chronic stroke survivors. Stroke.

[13] Studer B, Timm A, Sahakian BJ, Kalenscher T, Knecht S (2021). A decision-neuroscientific intervention to improve cognitive recovery after stroke. Brain.

[14] Park SH, Koh EJ, Choi HY, Ko MH (2013). A double-blind, sham-controlled, pilot study to assess the effects of the concomitant use of transcranial direct current stimulation with the computer assisted cognitive rehabilitation to the prefrontal cortex on cognitive functions in patients with stroke. J Korean Neurosurg Soc.

[15] Liu-Ambrose T, Eng JJ (2015). Exercise training and recreational activities to promote executive functions in chronic stroke: a proof-of-concept study. J Stroke Cerebrovasc Dis.

[16] Bunketorp-Käll L, Lundgren-Nilsson Å, Samuelsson H (2017). Long-term improvements after multimodal rehabilitation in late phase after stroke: a randomized controlled trial. Stroke.

[17] van de Ven RM, Murre JM, Veltman DJ, Schmand BA (2016). Computer-based cognitive training for executive functions after stroke: a systematic review. Front Hum Neurosci.

[18] Madhavan S, Shah B (2012). Enhancing motor skill learning with transcranial direct current stimulation - a concise review with applications to stroke. Front Psychiatry.

[19] Hordacre B, Moezzi B, Ridding MC (2018). Neuroplasticity and network connectivity of the motor cortex following stroke: a transcranial direct current stimulation study. Hum Brain Mapp.

[20] Dubovik S, Ptak R, Aboulafia T (2013). EEG alpha band synchrony predicts cognitive and motor performance in patients with ischemic stroke. Behav Neurol.

[21] Fernandez-Gonzalo R, Fernandez-Gonzalo S, Turon M, Prieto C, Tesch PA, García-Carreira Mdel C (2016). Muscle, functional and cognitive adaptations after flywheel resistance training in stroke patients: a pilot randomized controlled trial. J Neuroeng Rehabil.

[22] Sun R, Li X, Zhu Z, Li T, Li W, Huang P, Gong W (2021). Effects of combined cognitive and exercise interventions on poststroke cognitive function: a systematic review and meta-analysis. Biomed Res Int.

[23] Plant SE, Tyson SF, Kirk S, Parsons J (2016). What are the barriers and facilitators to goal-setting during rehabilitation for stroke and other acquired brain injuries? A systematic review and meta-synthesis. Clin Rehabil.

[24] Littooij E, Doodeman S, Holla J (2022). Setting meaningful goals in rehabilitation: A qualitative study on the experiences of clients and clinicians in working with a practical tool. Clin Rehabil.

